# A Δ-9 Fatty Acid Desaturase Gene in the Microalga *Myrmecia* *incisa* Reisigl: Cloning and Functional Analysis

**DOI:** 10.3390/ijms17071143

**Published:** 2016-07-16

**Authors:** Wen-Bin Xue, Fan Liu, Zheng Sun, Zhi-Gang Zhou

**Affiliations:** College of Fisheries and Life Science, Shanghai Ocean University, Shanghai 201306, China; xwb989510@gmail.com (W.-B.X.); liufanlflf@gmail.com (F.L.)

**Keywords:** RACE, Acyl-ACP desaturase, *Saccharomyces cerevisiae*, fatty acid composition

## Abstract

The green alga *Myrmecia incisa* is one of the richest natural sources of arachidonic acid (ArA). To better understand the regulation of ArA biosynthesis in *M. incisa*, a novel gene putatively encoding the Δ9 fatty acid desaturase (FAD) was cloned and characterized for the first time. Rapid-amplification of cDNA ends (RACE) was employed to yield a full length cDNA designated as *MiΔ9FAD*, which is 2442 bp long in sequence. Comparing cDNA open reading frame (ORF) sequence to genomic sequence indicated that there are 8 introns interrupting the coding region. The deduced MiΔ9FAD protein is composed of 432 amino acids. It is soluble and localized in the chloroplast, as evidenced by the absence of transmembrane domains as well as the presence of a 61-amino acid chloroplast transit peptide. Multiple sequence alignment of amino acids revealed two conserved histidine-rich motifs, typical for Δ9 acyl-acyl carrier protein (ACP) desaturases. To determine the function of *MiΔ9FAD*, the gene was heterologously expressed in a *Saccharomyces cerevisiae* mutant strain with impaired desaturase activity. Results of GC-MS analysis indicated that *MiΔ9FAD* was able to restore the synthesis of monounsaturated fatty acids, generating palmitoleic acid and oleic acid through the addition of a double bond in the Δ9 position of palmitic acid and stearic acid, respectively.

## 1. Introduction

Arachidonic acid (ArA, 20:4) is an essential fatty acid for humans. It acts as the precursor of various eicosanoids, whose imbalanced synthesis could lead to pathological conditions including asthma, ulcers and kidney diseases [1]. ArA is also known as a nutrient with great value. It makes contribution to the development of infant central nervous systems just like docosahexaenoic acid (DHA). Therefore, ArA has been suggested to be added to the baby’s milk formula as a key ingredient by FAO/WHO since 1995 [2].

So far, fish oil and porcine liver remain the main sources of ArA. The low contents of ArA (0.2%–0.5%), however, lead to a very high cost of production. In recent years, the exploration of microorganisms, such as microalgae as an alternative source of ArA has attracted huge interest [3,4,5]. The autotrophic oleaginous single cells of microalgae are able to convert simple minerals and enormous amounts of CO_2_ into biomass, which makes them ideal candidates for the commercialization of large-scale ArA production [6]. *Myrmecia incisa * Reisigl, a coccoid green alga belonging to Trebouxiophyceae, Chlorophyta [7], is among the richest sources of ArA. Under nitrogen starvation stress, its intracellular ArA content is up to 7% of the dry weight biomass [8], much higher than other microorganisms. However, the exact mechanism of ArA accumulation in *M. incisa* remains unclear. According to previous studies on biosynthesis of polyunsaturated fatty acids in eukaryotic algae [9,10,11], it has been generally recognized that the basic pathways are similar to those demonstrated in higher plants, which require a number of desaturases and elongases. Among the enzymes involved in lipid metabolism, Δ9 fatty acid desaturases (FADs) catalyze the first committed step. They add the first double bond to acyl chain between carbons 9 and 10, producing oleic acid (C18:1) and palmitoleic acid (C16:1) by desaturating stearic acid (C18:0) and palmitic acid (C16:0), respectively [12]. Δ9 FADs trigger the transition from saturated fatty acids to monounsaturated ones, which is the prerequisite to generate ArA. Therefore, the investigation on Δ9 FADs is of great value in elucidating the ArA biosynthesis in *M. incisa*. In this study, we aim to identify a novel gene encoding Δ9 FAD in *M*. *incisa* and determine its function in the host system. Previously we conducted the transcriptome analysis of *M*. *incisa* using 454 pyrosequencing and a total of 754,208 high-quality reads were obtained [13], which has laid a solid foundation for the present work. These findings will contribute to the knowledge about ArA synthesis and regulation, and may also benefit the genetic modification of *M*. *incisa* for an improved ArA production.

## 2. Results and Discussion

### 2.1. Cloning of Δ9 FAD Gene from M. incisa

Previously our research group has carried out pyrosequencing-based transcriptome analysis of *M. incisa* and a total of 754,208 high-quality reads were obtained. After clustering and assembly, these reads were assembled into 45,463 contigs and 54,780 singletons [13,14]. In the present study, three contigs were firstly targeted through the homology search and annotation, including Contig 16329 (1885 bp), Contig 3841 (1322 bp) and Contig 24483 (1210 bp). According to BlastX analysis, Contig 16329 exhibited the highest identity with known Δ9 FADs. Its encoded protein shared 72% similarity with *Mychonastes zofingiensis * (GeneBank ID: ACX71635), 72% with *Volvox carteri f. naqariensis* (GeneBank ID: XP_002949640), 71% with *Chlamydomonas reinhardtii * (GeneBank ID: XP_001691597) and 70% with *Haematococcus pluvialis* (GeneBank ID: ABP57425). Hence this contig was selected for further studies. Based on its sequence, RACE was employed to yield a full length cDNA named *MiΔ9FAD*, which was 2442 bp long in sequence (Figure S1). The coding region of the cDNA was revealed to contain a 1299-bp open reading frame (ORF). Upstream of the translation start codon is a 157-bp 5′-untranslated region (UTR), whilst between the stop codon and poly (A) tail is a 3′-UTR of 986-bp nucleotides.

**Figure 1:**
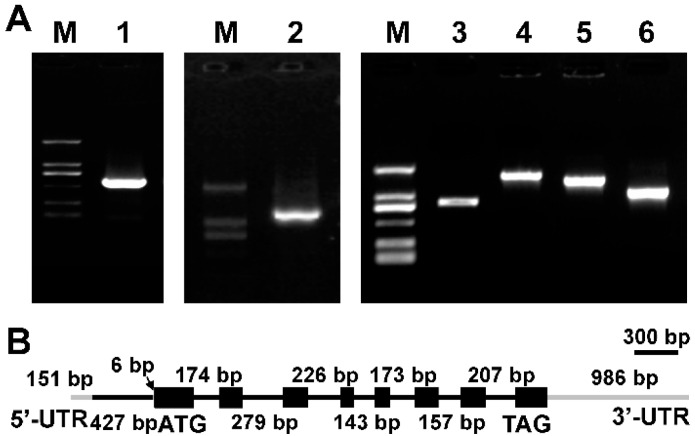
(**A**) Electrophoresis profiles of Δ9 FAD 5′-RACE and 3′-RACE product from *Myrmecia incisa*. M: D2000 marker; Lane 1: 5′-ends of Δ9 fatty acid desaturase (FAD); Lane 2: 3′-ends of Δ9 FAD; Lanes 3, 4, 5 and 6: the amplification products of Δ9 FAD using DNA as the template; (**B**) Schematic illustration of the gene structure of *MiΔ9FAD*. Black boxes: extrons; black lines: introns; gray lines: untranslated region (UTR).

Based on the cDNA sequence of *MiΔ9FAD* gene, its corresponding DNA sequence was cloned. Comparing cDNA ORF sequence to genomic sequence indicated that the full length of *MiΔ9FAD* DNA was of 4198 bp, and there were 8 introns interrupting the coding region. All of their splice sites conformed to the GT-AG rule (Figure 1).

### 2.2. Characterization of MiΔ9FAD

**Figure 2:**
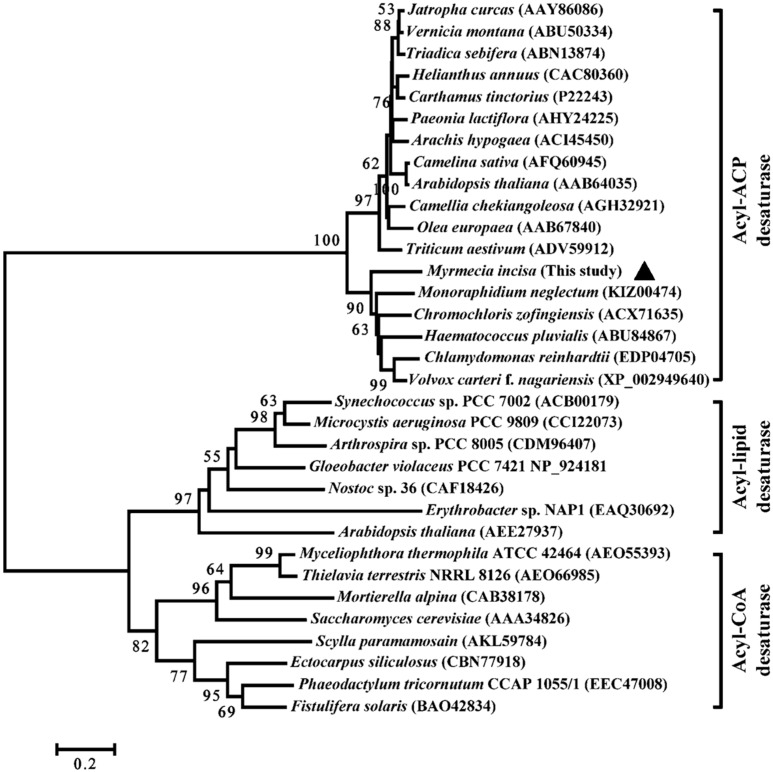
Phylogenetic tree of Δ9 FAD homology from various organisms. The brackets after species names indicated the GenBank ID of the enzymes. The predicted MiΔ9FAD in this study is marked by black solid triangle.

The deduced MiΔ9FAD protein is composed of 432 amino acids with a calculated molecular mass of 47.8 kDa and isoelectric point of 6.28. The phylogeny of the Δ9 FAD from microalgae as well as from animals, higher plants and bacteria was reconstructed using the neighbor-joining method. As shown in Figure 2, the predicted Δ9 FAD from *M. incisa* forms a much closer cluster with microalgae than higher plants and bacteria. All 33 Δ9 FAD protein sequences were separated into three groups, namely acyl-coenzyme A (CoA), acyl-acyl carrier protein (ACP) and acyl-lipid desaturases. These three Δ9 FAD members are present in various organisms: acyl-CoA desaturases are usually found in animal, yeast and fungal cells [15]; acyl-ACP desaturases are commonly found in higher plants and algae [16,17,18]; and acyl-lipid desaturases are mostly seen in plants and cyanobacteria [17,18]. Whilst three Δ9 FADs have different substrates to catalyze, all of them fulfill the same function. In this study, the predicted MiΔ9FAD was clustered into the acyl-ACP group with a bootstrap value of 100%, indicating it inserts the first double bond into fatty acids that are bond to ACP.

**Figure 3:**
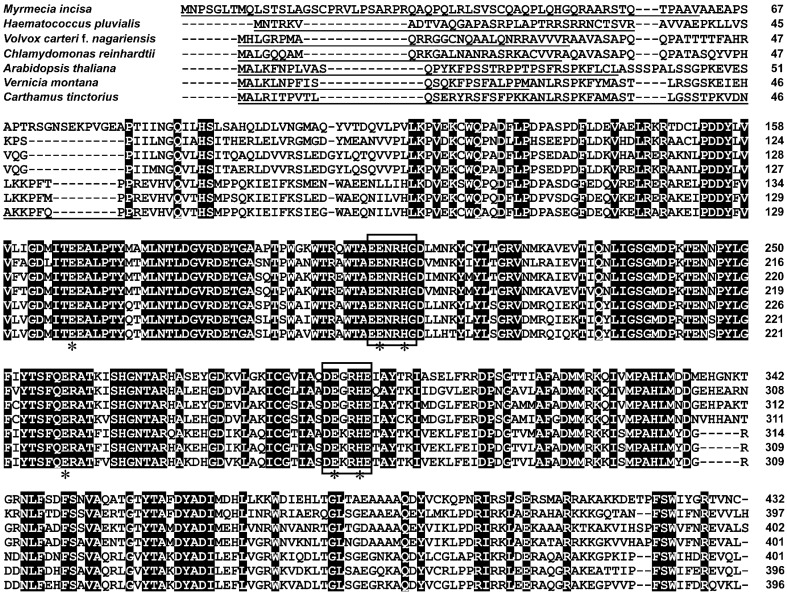
Sequence alignment of Δ9 FAD putative proteins from *Myrmecia incisa* and other organisms. The identical amino acid residues are shaded in black. Two conservative histidine-rich motifs are indicated by boxes. The ligands to the iron cluster are indicated by an asterisk. The chloroplast transit peptides are underlined.

According to TMHMM Server analysis, MiΔ9FAD had no transmembrane domains, whereas a 61-amino acid chloroplast transit peptide was found, and such chloroplast transit peptides were also present in Δ9 acyl-ACP desaturases from other organisms (Figure 3). Unlike acyl-CoA and acyl-lipid desaturases that are membrane bound, acyl-ACP desaturases are soluble and localized in the chloroplast [19]. It should be noted that the chloroplast transit peptide of MiΔ9FAD was excluded from its ORF in the following functional complementation assays. It is because the role of transit peptides is to direct the synthesized protein to a specific organelle, and once the transport is completed, they will be degraded by peptidases.

Multiple sequence alignment was conducted to search for conserved amino acid residues and sequence motifs. Results showed that MiΔ9FAD shared high identity to the published sequences of other algae and higher plants. In accord with previous reports [20,21], two conserved histidine-rich motifs were also identified, namely EENRHG and DEGRHE, which were located in residues 204–209 and 290–295 in MiΔ9FAD, respectively. Δ9 FADs belong to the class of di-iron-oxo proteins [22,23], and in this study, several ligands to the iron cluster were found, which play crucial roles for full enzymatic activity.

### 2.3. Functional Complementation Assays in S. cerevisiae

**Figure 4:**
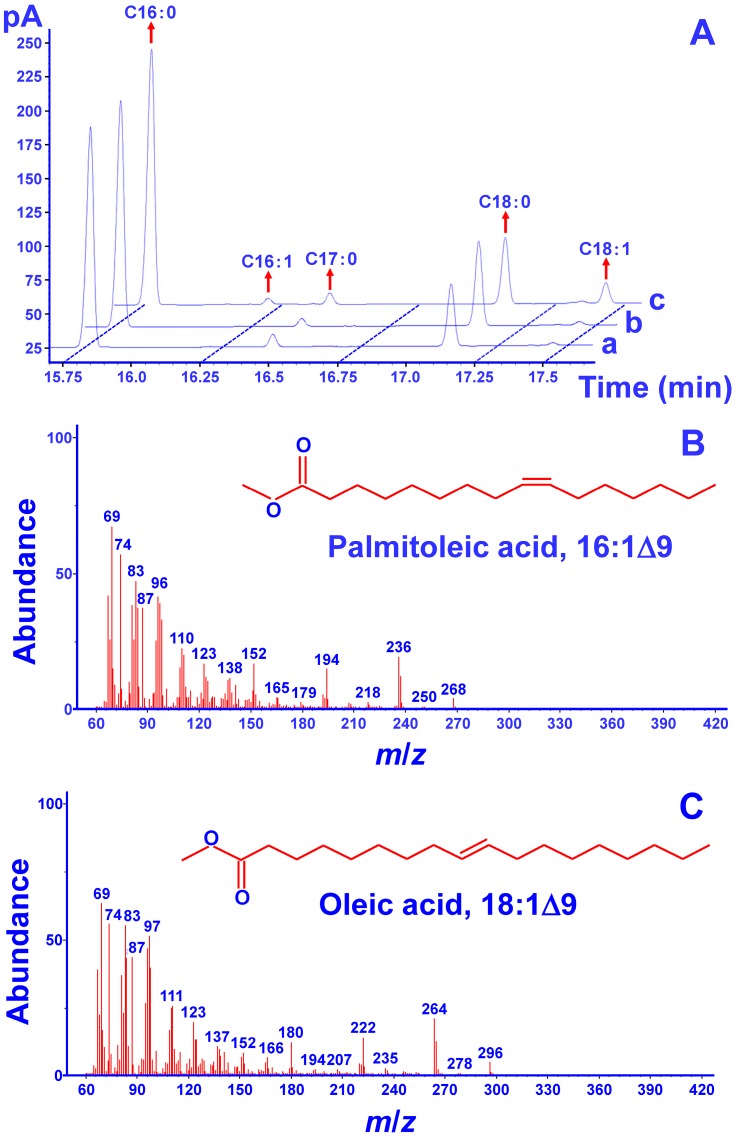
(**A**) Fatty acid profiles determined by gas chromatography (GC) analysis. (a) BY4389 mutant; (b) BY4389 transformed with empty pYES2; (c) BY4389 transformed with pY-MiΔ9FAD; (**B**,**C**) Profiles of two new fatty acids determined by mass spectrum analysis.

To verify the desaturation activity of MiΔ9FAD, a complementation assay was carried out in the mutant strain BY4389 of *S. cerevisiae*. This strain bears the mutation of *OLE1* gene, whose deprivation can lead to a complete abolishment of desaturase activity [24]. On the other hand, such functional disruption could be repaired by introducing the rat stearoyl-CoA desaturase gene [25]. The pYES2 vector was used, which contains the *URA3* gene for the selection of transformants on uracil-free minimal medium. The ORF of MiΔ9FAD was introduced into BY4389 to generate transgenic yeasts. Results of GC analysis (Figure 4A) indicated that pY-MiΔ9FAD led to two new fatty acids, whereas there were no additional peaks formed in the mutant strain carrying the empty vector. Mass spectrum analysis (Figure 4B,C) revealed that the new fatty acids were palmitoleic acid (C16:1) and oleic acid (C18:1), which were generated from the addition of a double bond in the Δ9 position of C16:0 and C18:0, respectively. These results indicated the complementation of unsaturated fatty acids-deficient phenotype, strongly supporting the desaturation activity of MiΔ9FAD.

In spite of the generation of C16:1 and C18:1, their contents were found to be very low. It could be because the substrates from *S. cerevisiae* did not perfectly match the Δ9 FADs from *M. incisa*. As MiΔ9FAD belongs to the acyl-ACP desaturase group, its favorable substrates are those saturated fatty acids bond to ACP. On the other hand, the fatty acids in the yeast are bond to CoA. Such incompatibility causes the efficiency of MiΔ9FAD to be reduced by up to 95% [26].

Δ9 FADs from different organisms may exhibit distinct substrate preference. For example, the Δ9 FAD from *Arabidopsis* [27] and *Psychrobacter urativorans * [28] tended to respectively catalyze C18:0 and C16:0. In the endosperm of coconut, the Δ9 acyl-ACP desaturase showed broad substrate specificity, targeting both saturated fatty acids [29]. In the present study, whilst the content of C16:0 was higher than C18:0 in *M. incisa*, the resulting C16:1 was much lower than C18:1. These results suggested that MiΔ9FAD may have a preference for C18:0. More detailed study needs to be carried out in future to verify it.

## 3. Materials and Methods

### 3.1. Algal Strain and Culture Conditions

*Myrmecia* *incisa* Reisigl H4301, originally obtained from the Culture Collection Algae of Charles University of Prague (CAPU), was the kind gift from Cheng-Wu Zhang, Ji’nan University, Guangzhou, China. The alga was cultivated in BG-11 medium [30] in 800-mL glass flasks, which were placed in a temperature-regulated photoincubator at 25 °C and illuminated from the side with a light:dark regime of 12 h:12 h by Phillips (Amsterdam, The Netherlands) cool-white fluorescent tubes (36 W) at a light irradiance of 115 µmol photons·m^−2^·s^−1^ [31]. During the cultivation, flasks were shaken several times per day at a regular interval. Algal cells were harvested at the late logarithmic growth phase by centrifugation at 5500 rpm for 10 min and washed three times with sterilized distilled water. Algal samples were stored in liquid nitrogen for total RNA and DNA extraction.

### 3.2. Yeast Strain and Culture Conditions

The *Saccharomyces cerevisiae* strain used in this study, BY4389 (His^−^, Leu^−^ and Ura^−^) was purchased from Osaka University, Osaka, Japan. This strain contains the *OLE1* mutation and therefore is defective in the synthesis of unsaturated fatty acids [24]. Before transformation, *S. cerevisiae* was grown in YPD medium containing 0.005% linoleic acid, 1% yeast extract, 2% peptone and 2% glucose on a shaker (220 rpm) at 28 °C. Prior to harvesting at late stationary phase (OD_600_ = 0.6–1.0), the culture was placed on ice for 15 min. Cells were collected by centrifugation at 5000 rpm for 5 min. The resulting pellet was washed three times with ice-cold sterilized water and then suspended in ice-cold sorbitol to give 1 × 10^10^ cells·mL^−1^. Aliquots of 0.1 mL of the cell suspension were dispensed to 1.5 mL micro-centrifuge tubes and then frozen at −80 °C for storage [32].

For functional expression in *S. cerevisiae* mutant strain, yeast cultures of transformants were grown on SC minimal medium lacking uracil (SC-U) according to the protocol (Invitrogen, Carlsbad, CA, USA). Before galactose induction, the medium was supplemented with 0.005% linoleic acid to allow the growth of yeasts. To induce the cells, linoleic acid was removed and galactose (2%, *w*/*v*) was added, and cells were further cultivated on a shaker (150 rpm) at 25 °C for 72 h. The procedure of centrifugation and storage was the same as above.

### 3.3. Genomic DNA and RNA Isolation

DNA of *M.* *incisa* was extracted using Plant Genomic DNA Extraction Kit (Tiangen Biotech, Beijing, China). Total RNA was extracted using TRIzol reagent according to the manufacturer’s instructions (Invitrogen, Carlsbad, CA, USA). The concentration of DNA and total RNA was determined spectrophotometrically by the ratio of absorbance at 260 and 280 nm.

### 3.4. Cloning of Δ9 FAD cDNA and Its Corresponding Gene

A putative *Δ9 FAD* was identified through searching against the transcriptome database of *M*. *incisa*, which was designated as *MiΔ9FAD*. First strand cDNA was synthesized from total RNA using PrimeScript™ RT Reagent Kit (TaKaRa, Dalian, China) following the manufacturer’s instruction. Based on the sequence of putative Δ9 FAD, specific primers (5′-1 forward, 5′-2 forward, and 3′-1 forward, Table 1) were designed for rapid amplification of 5′ and 3′ cDNA ends (RACE). SMART RACE cDNA Amplification Kit (Clontech, Mountain View, CA, USA) was used with the synthesized cDNA as template. RACE was performed according to the method described by Yu et al. [33]. Based on the cDNA sequence of MiΔ9FAD, its corresponding DNA sequence was cloned using the primer pairs listed in Table 1.

### 3.5. Bioinformatics Analysis

BioEdit 7.0, Clustal X and BLASTP were used for homologous sequences alignments with default parameter settings. ORF Finder (Available online: http://www.ncbi.nlm.nih.gov/gorf/gorf.html) was used to predict the coding region of Δ9 FAD. Spidey (Available online: http://www.ncbi.nlm.nih.gov/spidey/) was used to analyze the introns. ComputepI/MW (Available online: http://cn.expasy.org/tools/pi_tool.html) was used to calculate the isoelectric point and molecular weight. TMHMM Server v. 2.0 (Available online: http://www.cbs.dtu.dk/services/TMHMM-2.0/) was used to predict the transmembrane domains. Protein Prowler v. 1.2 (Available online: http://bioinf.scmb.uq.edu.au/pprowler_webapp_1-2/) was used to predict the subcellular localization. ChloroP 1.1 Server (Available online: http://www.cbs.dtu.dk/services/ChloroP/) was used to predict the chloroplast transit peptides. Smart (Available online: http://smart.embl-heidelberg.de/) was used to predict the key amino acids. Phylogenetic trees were constructed using MEGA 4.0 program with neighbor-joining (NJ) method [34].

### 3.6. Vector Construction and Heterologous Expression in S. cerevisiae

On the basis of the cloned cDNA of putative Δ9 FAD, the open reading frame (ORF) containing no chloroplast transit peptides was cloned with primers pY-F and pY-R (Table 1). The reaction system contained 1.0 µL cDNA, 1 µL primers, 10.5 µL RNase-free H_2_O and 12.5 µL 2× Taq PCR Master Mix. The reaction was performed in a gradient Mastercycler (Eppendorf, Hamburg, Germany) and programmed as follows: predenatured at 95 °C for 5 min, followed by 36 cycles consisting of denaturation at 94 °C for 1 min, annealing at 68.5 °C for 1 min and extension at 72 °C for 90 s, and ended by a final extension at 72 °C for 10 min. The PCR product was fractionated via 1.0% agarose gel electrophoresis, purified using Agarose Gel DNA Purification Kit Ver.2.0 (TaKaRa) and ligated into a pMD19-T vector (TaKaRa). The constructed vector was subsequently transformed into *Escherichia coli* DH5α competent cells (Biocolor BioScience & Technology Company, Shanghai, China), where positive clones were screened and verified by sequencing (Sangon, Shanghai, China). The resulting PCR products were digested with *Eco*RI and *Xba*I [35] and cloned into the corresponding sites of pYES2 vector (Invitrogen) by T4 DNA ligase, generating the recombinant expression vector pY-MiΔ9FAD. After the transformation into *E. coli* DH5α competent cells, positive clones were selected and verified as described above. The resulting recombinant expression vector and an empty pYES2 as the negative control were separately introduced into the *S. cerevisiae* mutant strain BY4389 by electroporation (Bio-Rad Laboratories, Hercules, CA, USA). Transformants were selected after the culture on SC-U agar plates containing 0.005% linoleic acid at 28 °C for 48–72 h.

### 3.7. Preparation of Fatty Acid Methyl Esters and GC-MS Analysis

Approximately 25 mg lyophilized yeast powder and 1 mL H_2_SO_4_-methanol solution (4%, *v*/*v*) were mixed in a test tube. After charging with nitrogen gas, the bottle was stirred and heated at 85 °C for 1 h. 1 mL distilled water and 1 mL hexane was added into the tube, mixed by vortexing and then centrifuged at 5500 rpm for 10 min. The supernatant was transferred into another tube, concentrated by bubbling nitrogen and stored at 4 °C for gas chromatography-mass spectrometry (GC-MS) analysis (Agilent Technologies, Wilmington, DE, USA).

The fatty acid methyl ester derivatives were subjected to GC-MS on a very polar column HP-88 (88%-cyanopropyl) aryl-polysiloxane (60 m × 250 μm × 0.2 μm) connected to a MS Engine quadrupole mass spectrometer. The column temperature was held at 70 °C for 1 min, increasing to 210 °C (held for 0 min) and 220 °C (held for 0 min) respectively at a rate of 10 °C·min^−1^, and then heated to 235 °C at a rate of 10 °C·min^−1^ and held for 8 min. The injection volume was 1 μL without split. Helium was used as the carrier gas at a flow rate of 0.6 mL·min^−1^, and the pressure was programmed at a constant flow mode. The mass spectrometer was operated in electron impact mode at ionization energy of 70 eV [36]. Fatty acids were identified based on the comparison of their mass spectra with those stored in NIST 08 MS libraries.

## 4. Conclusions

The high value of ArA and ArA-derived metabolites makes *M. incisa* an important model organism. Similar to other eukaryotic algae, the biosynthesis of ArA in *M. incisa* involves several desaturation and elongation steps, where the first committed step is catalyzed by Δ9 FAD. This is the first report describing Δ9 FAD in *M. incisa*, and findings of the present study may help better understand the regulation of ArA biosynthesis at molecular level, and contribute to developing a basis for an enhanced ArA content through genetic engineering.

## Figures and Tables

**Table 1:** Primers for *MiΔ9FAD* cloning and heterologous expression.

Primer	Sequence 5′-3′	Annealing Temperature/°C	Size of Amplificant/bp
**RACE for cDNA cloning**			
acp-1 Forward	CTGTCGGCCCACCAGTTAGA	–	–
acp-1 Reverse	GCCGTAGATCCAGGAGAAGG	60.9	1002
acp-5-gsp1	TCCTGGAAGGAGGTGTAGATGAAGC	66	–
acp-5-gsp2	GGCAGCACCTGGTCCGTAACATA	68	501
acp-3-gsp1	CCGCAACCTGTTTTCAGACTTCTCC	65	1230
**DNA cloning**			
D1-Forward	ACGCGGGGAGTGACAACACCAGCTGT	69.6	812
D1-Reverse	CTCTGAGTTGCCGCTCCTTGTGGGGG	–	–
D2-Forward	ATGAACCCGAGTGGGCTCACGATG	66.3	1542
D2-Reverse	GTCCATGCCAGAGCCAATCAGGTT	–	–
D3-Forward	ACAGCCGAGGAGAACCGTCATGGT	68.1	1376
D3-Reverse	CTAGCAGTTGACGGTGCGGCCGTAGAT	–	–
D4-Forward	GGACGAGACGCCCTTCTCCTGGAT	64.9	1005
D4-Reverse	GCCCTGCAGCGTTTTACAGCGC	–	–
**Heterologous expression in yeast**			
pY-Forward	cgGAATTCATGGCCGCAGAGGCCCCAT ^1,2^	68.5	1138
pY-Reverse	gcTCTAGACATCTAGCAGTTGACGGTGC ^1,2^	–	–

^1^ Lower case letters: base pairs added on the restriction enzyme recognition site; ^2^ Underlined letters: restriction sites.

## Supplementary Materials

Supplementary materials can be found at http://www.mdpi.com/1422-0067/17/7/1143/s1.
