# Supplementary material for: A Δ-9 Fatty Acid Desaturase Gene in the Microalga Myrmecia incisa Reisigl: Cloning and Functional Analysis
Source: Int J Mol Sci. 2016 Jul 16;17(7):1143. doi: 10.3390/ijms17071143 (PMC4964516; doi:10.3390/ijms17071143)
Supplement: Supplementary file 1 [file ijms-17-01143-s001.pdf]

Supplementary Materials: A Δ-9 Fatty Acid Desaturase Gene in the Microalga *Myrmecia incisa* Reisigl: Cloning and Functional Analysis

Wen-Bin Xue, Fan Liu, Zheng Sun and Zhi-Gang Zhou

ACGCGGGGAGTGACAACACCAGCTGTCCAACGAAACCTAGTATTCTTT
GTGCACAGCAAACGACTGCCTGTACGTCGTGAAACGCATAGCCCCTTC
TAAACTTCTCGACAGCCCCCATCTCTCAAGAACCCGGACAGTCACTGG
CAACCAGGGGCAGATGAACCCGAGTGGGCTCACGATGCAGCTCAGCA
CCAGCCTTGCAGGCAGCTGTCCCAGAGTCCTACCTTCAGCACGCCCTA
GGCAAGCCCAGCCGCAGCTTCGCCTCAGCGTCTCCTGCCAGGCACAGC
CGCTGCAGCATGGCCAGCGCGCAGCTCGAAGCACGCAAACCCCTGCA
GCCGTTGCCGCAGAGGCCCCATCTGCCCCCACAAGGAGCGGCAACTCA
GAGAAGCCTGTTGGCGAGGCGCCAACGATCATCAATGGCCAGATCTTG
CACAGCCTGTCGGCCCACCAGTTAGACCTGGTCAACGGGATGGCGCAG
TATGTTACGGACCAGGTGCTGCCTGTGCTCAAGCCCGTGGAGAAGTGC
TGGCAGCCCGCAGACTTCCTGCCCGACCCCGCATCCCCGGACTTCCTG
GACGAGGTGGCAGAGCTCCGGAAGCGCACCGACTGCCTACCGGATGA
CTACCTGGTGGTGCTCATTGGGGACATGATCACGGAGGAGGCGCTCCC
CACATACATGGCCATGCTCAACACGCTGGACGGTGTGCGTGACGAGAC
AGGCGCCGCACCGACGCCTTGGGGCAAGTGGACCCGCCAGTGGACAG
CCGAGGAGAACCGTCATGGTGACCTCATGAACAAGTACTGCTACCTGA
CAGGGCGTGTCAACATGAAGGCGGTGGAGGTGACAATCCAGAACCTG
ATTGGCTCTGGCATGGACCCCAAGACGGAGAACAACCCCTACCTGGGC
TTCATCTACACCTCCTTCCAGGAGCGTGCGACAAAGATCTCGCACGGG
AACACCGCACGCCATGCCAGCGAGTATGGCGACAAGGTGCTGGGCAA
GATCTGCGGCGTAATCGCACAGGATGAGGGCCGGCACGAGATCGCCT
ACACGCGCATTGCGTCAGAGCTGTTCAGGCGAGACCCGTCCGGCACCA
CGATTGCGTTCGCAGACATGATGCGCAAGCAGATTGTGATGCCCGCCC
ACCTGATGGACGACATGGAGCACGGCAACAAGACGGGCCGCAACCTG
TTTTCAGACTTCTCCAACGTGGCGCAGGCCACGGGCACCTACACAGCC
TTCGACTACGCGGACATCATGGACCACCTGCTGAAGAAGTGGGACATT
GAGCACCTGACGGGCCTGACGGCCGAGGCCGCCGCGGCGCAGGACTA
CGTGTGCAAGCAGCCCAACCGCATCCGCAGCCTGTCGGAGCGCAGCAT
GGCGCGCCGCGCCAAGGCCAAGAAGGACGAGACGCCCTTCTCCTGGA
TCTACGGCCGCACCGTCAACTGCTAGATGTGGGCTGTCGCATGTGGAA
GTGCTTGCCAGACTGGCCGCACTGTTGACTGCTGGATGCGCCATGGCG
CATATGGAGTGCTTGTCACACCCGTGCCGCATCATGCACAGCCATGTG
CCTGGTATACAAGCCAAGGACCCTGCGGGAGTGTGATGGGGCTTGCTT
CTCAAGCCCCTGAGTGTGTGAGGATCGTGTTTGAGGCTCAGCAATGGC
ATAGGCTGATGGGACGTCATTCACTTGCTCGCTAGGTAAGGGAGCACA
CGGCAGGTGGCTGATTTGTCATGAGGTTCAGGGCAGCATGTCACCGGT
CGGTCAGGTGGATGAGGTTGCAAAGTTGGAAAAATCCATGCTGGGTGT
GGATTGCTTCGGTCAGTCATGTTGCTGGGCTGCAACACATGTGTCAGC
TCCTACAGCTACCCATCATTCTTGCCTGTTGTCGCGTGTTCGGCCAGGC
CATACCTTGTTCAGGCGCCCTGTGTGTGCAAGTCTGTGCTACACCCTTT
TAGGAACATATTGCACTCCATGGGTATGGCAGGCTAGATGGCATGGCA
ACTCAAAGAGGCAATCAAAGAACTGGGCAGTATATACCATTATTTGCT
CCCCTGTGTGGCTGTTGGCATTCTGATTTAGGCCTGTGACTTGCAACCA
GCCAGGGGCGGTGCAAGATATTCCATGTGAGTGGCAGATTAAGCATGT
TGGTGATGATTGTGCAGAATGACTGATGGAAGTCGACATTGCATGCGA
GCGGCCATGGTGTCGCATCTGTATGAGAGTGAGAGCGGTAGGATCAA
GAAGTGCTTGTACAACTAGCGACACTAGATACAGTGGCTCCATGGTGG
TGGTTGACATACGCCTCCTTGGCAGCCTCGGGCGGTGGTATTGAGAAC
GTTGGTCCACATCCAGGGTCCACCTGGATTTGTAAGTACAGTGCGCGC
GCTGTAAAACGCTGCAGGGCAAAAAAAAAAAAAAAAAAAAAAAAAA
AAAA

Figure S1. The full length cDNA sequence of *MiΔ9FAD.* The predicted chloroplast transit peptides are underlined. The initiation codon and termination codon are in boxes.
